# p53 Is Active in Human Amniotic Fluid Stem Cells

**DOI:** 10.1089/scd.2017.0254

**Published:** 2018-10-25

**Authors:** Melissa Rodrigues, Ivana Antonucci, Seham Elabd, Shilpa Kancherla, Marco Marchisio, Christine Blattner, Liborio Stuppia

**Affiliations:** ^1^ Laboratory of Molecular Genetics, Department of Psychological, Health and Territorial Sciences, School of Medicine and Health Sciences, G. d' Annunzio University, Chieti-Pescara, Italy.; ^2^ Institute of Toxicology and Genetics, Karlsruhe Institute of Technology, Karlsruhe, Germany.; ^3^ Centre of Aging Science and Translational Medicine (Ce.S.I.-Me.T.), G. d'Annunzio University, Chieti-Pescara, Italy.; ^4^ Department of Human Physiology, Medical Research Institute, Alexandria University, Alexandria, Egypt.; ^5^ Department of Medicine and Aging Sciences, School of Medicine and Health Sciences, G. d' Annunzio University, Chieti-Pescara, Italy.

**Keywords:** amniotic fluid cells, p53, proliferation, DNA damage response, apoptosis, differentiation

## Abstract

Despite increasing interest in human amniotic fluid cells, very little is known about the regulation and function of p53 in this cell type. In this study, we show that undifferentiated human amniotic fluid cells express p53, yet at lower levels than in cancer cells. The p53 protein in amniotic fluid cells is mainly localized in the nuclei, however, its antiproliferative activity is compromised in these cells. *Igf2*, a maternal imprinted gene, and *c-jun*, a proto-oncogene, are regulated by p53 in these cells. DNA damage leads to an increase in p53 abundance in human amniotic fluid cells and to transcriptional activation of its target genes. Interestingly, cell differentiation toward the neural lineage leads to p53 induction as differentiation progresses.

## Introduction

Almost four decades ago, p53 was identified and ever since has remained at the core of cancer research. As a crucial protein in multicellular organisms, it is described as a “cellular gatekeeper” or “guardian of the genome” because of the prominent role it plays in preserving genomic stability [1–3]. p53, which is encoded by the *tp53* gene, is involved in a variety of cellular processes, including proliferation, senescence, differentiation, apoptosis, ferroptosis, DNA repair, metabolism, angiogenesis, and autophagy [4,5].

Functionally, p53 is a transcription factor that elicits its cellular functions mostly through transcriptional activation of target genes. Besides its primary function as a transcription factor, p53 can also promote apoptosis through direct interaction with proapoptotic and antiapoptotic proteins [6].

The activity of p53 is always under tight control, which ensures that it is not overly abundant in nonstressed cells. Apart from all the activities it plays in adult somatic cells, p53 seems to be involved in the self-renewal of embryonic stem (ES) cells and other adult stem cells, as well as in the onset of differentiation [7]. In adult stem cells like neural or hematopoietic stem cells, p53 negatively regulates proliferation and self-renewal, and helps to maintain their quiescent state [8,9].

Human amniotic fluid cells, ordinarily discarded as medical waste, present potentially a novel source for therapeutically used stem cells. These human amniotic fluid stem (hAFS) cells are in an intermediate state between pluripotent ES cells and lineage-restricted adult progenitor cells [10]. The population of hAFS cells is highly heterogeneous and they exhibit a high proliferation rate and wide differentiation potential, including differentiation into hematopoietic, neurogenic, osteogenic, chondrogenic, adipogenic, renal, and hepatic lineages [11,12]. Most intriguingly, unlike ES cells, hAFS cells do not produce teratomas when transplanted into nude mice [13]. This important attribute along with their high genomic stability and epigenetic fidelity makes hAFS cells an ideal candidate for stem cell-based therapeutic applications.

Recently it has become more evident that apart from the role that p53 plays as a tumor suppressor, it is an important modulator of stem cell fate. Loss or functional defects in its activity can lead to implications like tumor formation or genomic instability. Despite the increasing interest in hAFS cells, very little is known about the regulation and function of p53 in this cell type.

In this article, we present that p53 is expressed and mainly localized in the nucleus of hAFS cells. The antiproliferative activity of p53 is compromised under nonstressed conditions in these cells, but p53 becomes active during the DNA damage response. We also show that the insulin-like growth factor 2 gene (*igf2*), a maternally imprinted gene that is related to cell development and proliferation, and *c-jun*, an important proto-oncogene, are regulated by p53 in hAFS cells.

## Materials and Methods

### Cell lines and their treatment

The study has been approved by the ethics committee of the G. d'Annunzio University of Chieti-Pescara, ASL Lanciano-Chieti-Vasto, Italy. A sample of amniotic fluid was taken after informed written consent from a woman at 18 weeks of pregnancy at the Cytogenetics Laboratory, G. d'Annunzio University of Chieti-Pescara. Two milliliters of the amniotic fluid were centrifuged at 1,200 rpm for 5 min and the pellet was used to establish the cell line (hAFS cells). All the experiments were done using a hAFS cell line from one donor, to maintain linearity throughout.

hAFS cells were cultured in Iscove's modified Dulbecco's medium (Life Technologies), supplemented with 20% fetal bovine serum (FBS; Life Technologies), 100 U/mL penicillin, 100 μg/mL streptomycin (Life Technologies), 2 mM l-glutamine (Life Technologies), and 5 ng/mL basic fibroblast growth factor 2 (Thermo Fisher Scientific), and incubated at 37°C in a humidified atmosphere with 5% CO_2_. The medium was changed every 2 days.

For differentiation, cells were cultured as reported earlier [14,15] in Dulbecco's modified Eagle medium (DMEM; Life Technologies) supplemented with 10% FBS, 0.1 mM β-mercaptoethanol (Life Technologies), 1% penicillin/streptomycin, and 1 μM all-trans-retinoic acid (Sigma-Aldrich) for up to 28 days. The medium was changed every 2 days.

MCF-7 cells (human breast adenocarcinoma cells), HCT-116 cells (human colon cancer cells), U2OS cells (human osteosarcoma cells), A547 cells (human lung carcinoma cells), and H1299 cells (human nonsmall cell lung carcinoma cells) were cultured in DMEM supplemented with 10% FBS and 1% penicillin/streptomycin at 37°C with 5% CO_2_ in a humidified atmosphere.

hAFS cells and H1299 cells were transfected with PromoFectin (PromoKine) according to the manufacturer's recommendation and incubated for 24 h. siRNA transfections were performed using RiboJuice™ (Millipore) according to the manufacturer's recommendation. The medium was changed after 48 h, and cells were harvested after 72 h. Sequences of siRNAs are available on request.

To induce DNA damage, cells were treated with 50 μM (f.c.) Etoposide (Sigma-Aldrich). Pifithrin-α (Sigma-Aldrich) was used at a final concentration of 10 μM.

### Plasmids and antibodies

Plasmids used were the mammalian expression vector pcDNA3 (Invitrogen) and pcDNA3 containing the cDNA of p53 (pcDNA3-p53) that has been described previously [16].

Primary antibodies against p53 (Millipore) diluted 1:5,000, P21 (BD Biosciences) diluted 1:1,000, Mdm2 (Millipore), Nestin (BD Biosciences) diluted 1:1,000, MdmX (Sigma-Aldrich) diluted 1:4,000, poly ADP-ribose polymerase (PARP) (BioLegend) diluted 1:1,000, MAP2 (Cell Signaling Technology) diluted 1:1,000, β-tubulin III (Abcam) diluted 1:2,000, β-actin (Santa Cruz Biotechnology) diluted 1:1,000, and glyceraldehyde 3-phosphate dehydrogenase (Hytest) diluted 1:20,000 were used. Horseradish peroxidase-conjugated anti-mouse and anti-rabbit immunoglobulin G (IgG) (DAKO) diluted 1:2,000 were used as secondary antibodies. All antibodies were diluted in complete cell culture medium.

### Sodium dodecyl sulfate–polyacrylamide gel electrophoresis and western blotting

Cells were washed twice with phosphate-buffered saline (PBS), scraped into PBS, pelleted at 1,200*g* for 2 min, and lysed in NP-40 lysis buffer (150 mM NaCl, 50 mM Tris [pH 8], 5 mM EDTA, 1% NP-40, and 1 mM phenylmethylsulfonyl fluoride) for 10 min on ice. The protein extract was cleared by centrifugation at 13,000*g* at 4°C for 15 min and the protein concentration of the supernatant (protein extract) was determined by the method of Bradford.

Forty micrograms of total protein (unless otherwise indicated) were heated to 95°C for 10 min in 2 × sample buffer (2% sodium dodecyl sulfate [SDS], 80 mM Tris [pH 6.8], 10% glycerol, 5% 2-mercapthoethanol, and 0.001% bromophenol blue), separated on an SDS-polyacrylamide gel, and transferred onto a polyvinylidene difluoride membrane (Millipore). The membrane was blocked for 1 h in 5% dry milk diluted in 0.2% Tween 20 in PBS before incubation with primary antibodies. Primary antibodies were incubated overnight at 4°C, followed by three 5-min washes with PBS—0.2% Tween 20. The membrane was incubated for 60 min with a secondary antibody and given three 5-min washes with PBS—0.2% Tween 20. The western blots were developed by the enhanced chemiluminescence method.

### MTT-assay

Cells were plated at a concentration of 100,000 cells per well in a 12-well plate and transfected with siRNA targeted against p53 and control siRNA that is not directed against any known gene. Ninety-six hours after plating, 3-1-2,5-diphenyltetrazolium bromide (MTT) was added to a final concentration of 0.2 mg/mL and incubated for 3 h. Afterward the medium was removed, cells and the formazan salt were solubilized with isopropanol and the absorbance was determined at λ 550 nm.

### Immunofluorescence staining

hAFS cells were grown in two-chamber slides at a concentration of 50,000 cells per chamber for 24 h. After washing with PBS, cells were fixed with 4% paraformaldehyde for 20 min at 37°C, chilled on ice for 1 min, and incubated for 30 min on ice with ice-cold 90% methanol solution prepared with distilled water. For the immunofluorescence staining after differentiation, hAFS cells were grown up to 28 days on coverslips in a differentiation medium with a change of medium every 2 days. After washing with PBS, cells were fixed on ice for 8 min with an ice-cold methanol-acetone solution (1:1) and washed with PBS.

After washing with an incubation buffer (50 mM Tris, 150 mM NaCl, 0.1% Tween 20, and 5% BSA), cells were incubated overnight with primary antibodies (DO-1, γ-H2AX, and glial fibrillary acidic protein (GFAP), diluted 1: 500; MAP2, diluted 1:200, and β-tubulin III, diluted 1 μg/mL in the incubation buffer). The cells were washed twice with the incubation buffer and incubated for 2 h at room temperature in the dark with an antibody directed against mouse IgG coupled to Alexa-Fluor-488 (Life Technologies) or against rabbit IgG coupled to Alexa-Fluor-546 (Life Technologies) diluted 1:1,000 in the incubation buffer. After washing with PBS, the slides were incubated with 4′,6-diamidino-2-phenylindole (f.c. 1 mg/mL prepared in PBS) in the dark for 45 min, washed again with PBS, and mounted on microscope slides with Hydromount (Polysciences, Inc.). Cells were analyzed using a Leica DMI4000B microscope.

### Real-time polymerase chain reaction

Cells were suspended in Trifast (PeqGold; VWR Life Sciences), vortexed, and incubated for 5 min. Chloroform was added and the mixture was incubated for 5 min. The mix was centrifuged at 13,000*g* for 5 min at 4°C, then mixed with isopropanol and 2 μL Roti-Pink (Roth) to improve precipitation of the RNA, and placed on ice for 10 min, followed by centrifugation at 13,000*g* for 10 min at 4°C. The pellet was washed twice with 75% ethanol at 13,000*g* for 10 min and suspended in 30 μL RNAase-free water. The RNA solution was treated with DNaseI and transcribed into cDNA using random primers and RevertAid H MinusM-MuLV reverse transcriptase (Promega).

Real-time polymerase chain reaction was performed with GoTaq^®^ qPCR Master Mix (Promega). The cDNA was denatured for 15 min at 95°C followed by 40 cycles of 95°C for 15 s and 60°C for 1 min using the 7000 ABI sequence detection system and gene-specific primers. The signals were normalized to the signals for β-actin. Sequences of primers are available on request.

### Statistics

Statistical comparisons between two groups were made using Student's *t-*test.

## Results

### Phenotypic characterization of the hAFS cells

The hAFS cell line that was used for this study was first tested for different surface and intracellular markers to ascertain that the cells are indeed in an intermediary state between pluripotent ES cells and lineage-restricted adult progenitor cells [10]. As previously reported [17,18], the hAFS cells did not show surface expression of hematopoietic surface markers (eg, CD14, CD34, and CD45), but expressed a variety of established mesenchymal markers (eg, CD73, CD90, and CD105), several related surface adhesion molecules (eg, CD29, CD44, CD146, and CD166), and the stemness markers hTERT, Sox-2, Oct3/4, and SSEA-4. The hAFS cells, however, did not express CD117 and CD133 (Supplementary Fig. S1 and Supplementary Table S1; Supplementary Data are available online at www.liebertpub.com/scd).

### p53 abundance and localization

**FIG. 1.**
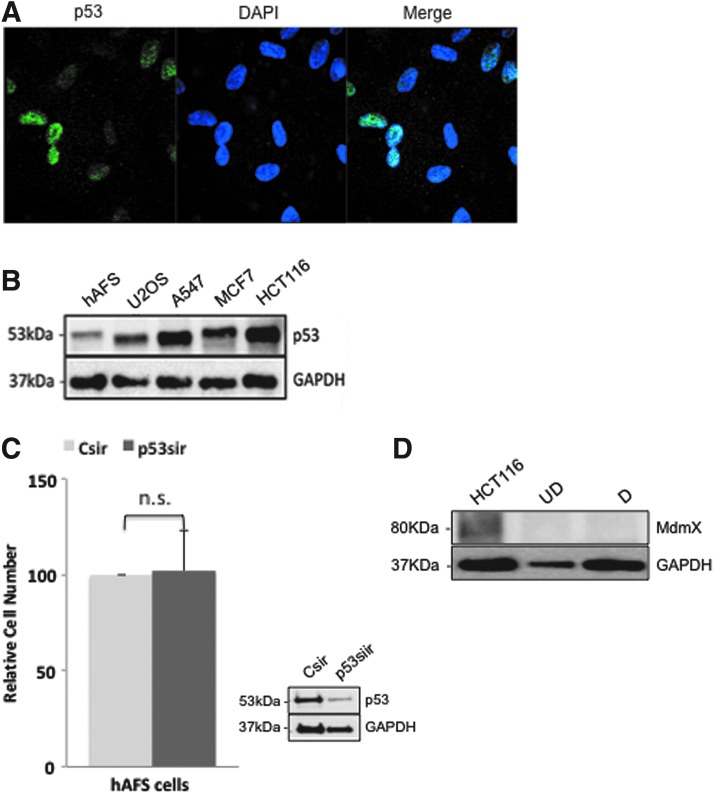
p53 localization, levels, and activity in hAFS cells. **(A)** hAFS cells grown in chamber slides were fixed and stained for p53 with the anti-p53 antibody DO-1 (*green*). The cell nucleus was stained with DAPI (*blue*). **(B)** hAFS cells, U2OS cells, A549 cells, MCF7 cells, and HCT116 cells were harvested and protein lysates were prepared. Forty micrograms of protein lysate were loaded onto an SDS-PAGE gel and hybridized for the detection of p53. GAPDH was used as loading control. **(C)** hAFS cells were transfected in duplicates with a siRNA targeted against p53 (p53sir) or with a control siRNA (Csir) and incubated for 96 h. One of the duplicates was used to determine the relative amounts of cells by MTT assay. The graph shows mean values and standard deviations of three independent experiments. The other duplicate was used to monitor the abundance of p53 by western blotting. Hybridization with GAPDH was performed for loading control. **(D)** Undifferentiated hAFS cells (UD) and cells differentiated with retinoic acid for 7 days (D) were harvested and protein lysate was prepared. Protein lysate of HCT116 cells was used as positive control. Forty micrograms of protein were loaded onto an SDS-PAGE gel and MdmX abundance was monitored by western blotting. GAPDH was used as loading control. DAPI, 4′,6-diamidino-2-phenylindole; GAPDH, glyceraldehyde 3-phosphate dehydrogenase; hAFS, human amniotic fluid stem; n.s., not significant; SDS-PAGE, sodium dodecyl sulfate–polyacrylamide gel electrophoresis. Color images available online at www.liebertpub.com/scd

As a transcription factor, p53 needs to be nuclear to be active. However, in earlier reports about ES cells, p53 was found to be cytoplasmic [19,20]. To determine the localization of the p53 protein in hAFS cells, we performed immunofluorescence staining and found that in hAFS cells, the majority of p53 is localized in the nucleus. Similar to murine ES cells [14], we observed in hAFS cells that the distribution of p53 was quite heterogeneous with some cells expressing high amounts of p53 and most cells expressing low amounts (Fig. 1A).

In mesenchymal stem cells (MSCs), both a constant expression during long-term cultivation and downregulation during the first 15 passages have been reported for p53 [21–23]. Pertaining to these studies, we investigated p53 abundance at early and late passages of hAFS cells. As shown in Supplementary Fig. S2, we observed a stable expression of the p53 protein that did not change with increased passage number (Supplementary Fig. S2).

hAFS cells possess intermediate properties between pluripotent ES cells and lineage-restricted adult progenitor cells [10]. The abundance of p53 in murine ES cells is much higher than in cancer cells, while its abundance in human ES cells is comparable with cancer cells [24–27]. These differences in p53 abundance in different ES cells and differentiated cells made us wonder to which level p53 is expressed in hAFS cells.

Because of the species specificity of the p53 antibodies and the inability to work with human ES cells, we could not directly compare the abundance of p53 in murine and human ES cells and hAFS cells. We therefore decided to compare the levels of p53 in hAFS cells with different tumor cell lines. For the different cell lines, we chose U2OS, HCT116, A549, and MCF7. All these cell lines express wild-type p53 and have therefore relatively low p53 levels [28]. We prepared protein lysates of all these cell lines and of hAFS cells and compared the abundance of the p53 protein by western blotting. While p53 expression in the different tumor cell lines was relatively comparable, hAFS cells expressed much lower levels of p53 (Fig. 1B).

### The antiproliferative activity of p53 is suppressed in hAFS cells

Expression of p53 usually decreases cell proliferation [5]. However, since hAFS cells are an intermediate between stem cells and more differentiated progenitor cells, [10] and since it has been shown that in murine ES cells, p53's antiproliferative activity is compromised [14], we wondered whether p53 is able to suppress cell proliferation in hAFS cells. To investigate this conjecture, we downregulated p53 in hAFS cells and monitored the increase in cell number with an MTT assay. Most interestingly, we only observed a slight difference in the rate of proliferation in control cells and in cells with downregulated p53 (Fig. 1C). This small difference was far beyond the difference that others have seen, for example, in murine embryonic fibroblasts [29]. This result indicates that the antiproliferative activity of p53 is compromised in hAFS cells similar to ES cells [14].

The most well-known negative regulators of p53 are Mdm2 and MdmX, which associate with the transactivation domain of p53 [30,31]. In ES cells, the antiproliferative activity of p53 is controlled at least, in part, by high amounts of MdmX [14]. Since we found the antiproliferative activity of p53 compromised in hAFS cells, we asked whether MdmX might also be present in high amounts in hAFS cells and monitored MdmX abundance in hAFS cells by western blotting. To see whether MdmX abundance might eventually change during differentiation as it does in murine ES cells [14], we analyzed both undifferentiated and differentiated hAFS cells. For positive control, we used HCT116 cells that express relatively high levels of MdmX [32]. As shown in Fig. 1D, expression of MdmX was below the detection limit both in the undifferentiated and differentiated hAFS cells (Fig. 1D).

### p53 regulates the expression of *igf2* and *c-jun* in hAFS cells

As p53 is abundantly expressed in hAFS cells, but does not suppress cell proliferation in unstressed hAFS cells, we wondered whether it might eventually control transcription of noncanonical target genes of p53 as it has been observed in murine ES cells [14]. We therefore analyzed expression of *igf2* and *c-jun* that were induced by p53 in murine ES cells, but downregulated in differentiated cells [14,33]. To test this possibility, we downregulated p53 in hAFS cells by transfecting a siRNA targeted against p53. For control, we transfected a control siRNA that is not directed against any known gene. Seventy-two hours after transfection, we harvested the cells and monitored expression of *c-jun* and *igf2* by quantitative real time-polymerase chain reaction (qRT-PCR).

**FIG. 2.**
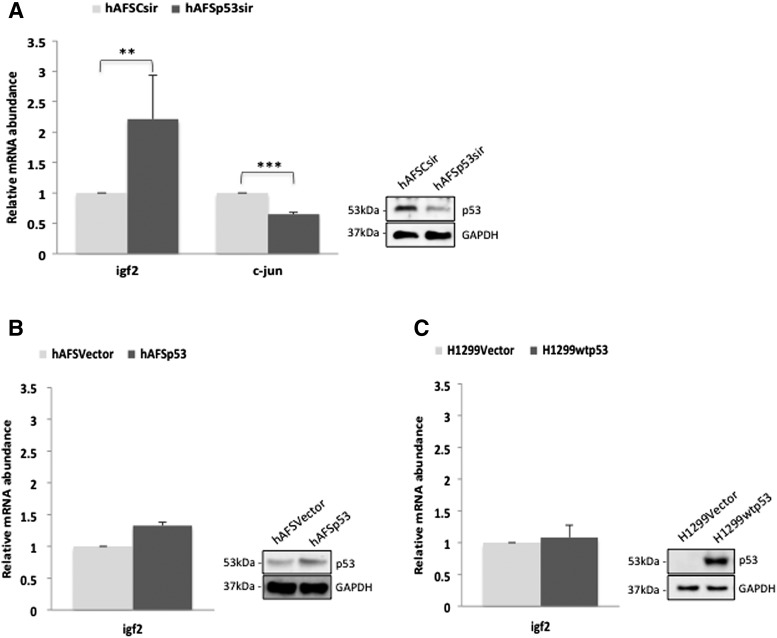
p53 regulates the expression of *igf2* and *c-jun* in hAFS cells. **(A)** hAFS cells were transfected in duplicates with a siRNA targeted against p53 (hAFSp53sir) or with control siRNA (hAFSCsir). Seventy-two hours after transfection, cells were harvested. From one of the duplicates, RNA was prepared, transcribed into cDNA, and analyzed by qRT-PCR. Abundance of specific cDNAs was normalized by determining the abundance of the housekeeping gene β-actin. Relative abundance of the specific RNA in the cells with control siRNA was set to one. The graph shows mean values and standard deviations of four independent experiments (**P < 0.02; ***P < 0.005). The second duplicate of the cells was used to monitor p53 abundance by western blotting. **(B)** hAFS cells (with a female karyotype) were transfected with a plasmid encoding p53 (hAFSp53) or with vector DNA (hAFSVector). The relative abundance of igf2 mRNA was monitored by qRT-PCR as described in the legend to part A. The graph shows mean values and experimental variation of two independent experiments. A second duplicate was used to monitor p53 abundance by western blotting. **(C)** H1299 cells were transfected with a plasmid encoding wild-type p53 (H1299wtp53) or with vector DNA (H1299Vector). The relative amount of igf2 mRNA was monitored by qRT-PCR as described in the legend to part A. The graph shows mean values and experimental variation of two independent experiments. A second duplicate was used to monitor p53 abundance by western blotting. *igf2,* insulin-like growth factor 2 gene; qRT-PCR, quantitative real time-polymerase chain reaction.

As shown in Fig. 2A, expression of *c-jun* was reduced in hAFS cells when p53 was downregulated, consistent with the report on murine ES cells [14] (Fig. 2A). Most interestingly, expression of *igf2* was strongly increased after downregulation of p53 in hAFS cells (Fig. 2A). This was in contrast to ES cells where expression of *igf2* was reduced when p53 was absent [14]. To further confirm that *igf2* expression is indeed increased by p53 in hAFS cells, we overexpressed p53. However, to our surprise, *igf2* mRNA levels remained largely the same when p53 was overexpressed (Fig. 2B). To see whether there is a difference between the regulation of *igf2* by p53 in hAFS cells and cancer cells, we overexpressed p53 in the p53-negative cell line H1299. Here, *igf2* levels changed marginally when p53 was transfected (Fig. 2C).

*Igf2* is a maternally imprinted gene that is regulated in a gender-specific manner [34]. Since *igf2* was regulated by p53 in hAFS cells, we wondered whether p53 might be involved in the gender-specific regulation of *igf2.* As the cells, where we have found an induction of *igf2* by p53 (Fig. 2B), had a 46XX karyotype, we also overexpressed p53 in hAFS cells with a XY karyotype and aimed at monitoring the expression of *igf2* also in this cell line. However, in the hAFS cell line with a XY karyotype, *igf2* mRNA levels were below the detection limit.

### p53 is induced during differentiation in hAFS cells

p53 has been shown to be involved in the differentiation of ES and adult stem cells [4,35,36]. To investigate if p53 is also involved in the differentiation of hAFS cells, we differentiated the cells using all-*trans* retinoic acid for several days and monitored their morphological changes under the microscope followed by immunostaining with several neuronal markers. We observed drastic changes in the morphology of hAFS cells after induction of differentiation (Supplementary Fig. S3).

**FIG. 3.**
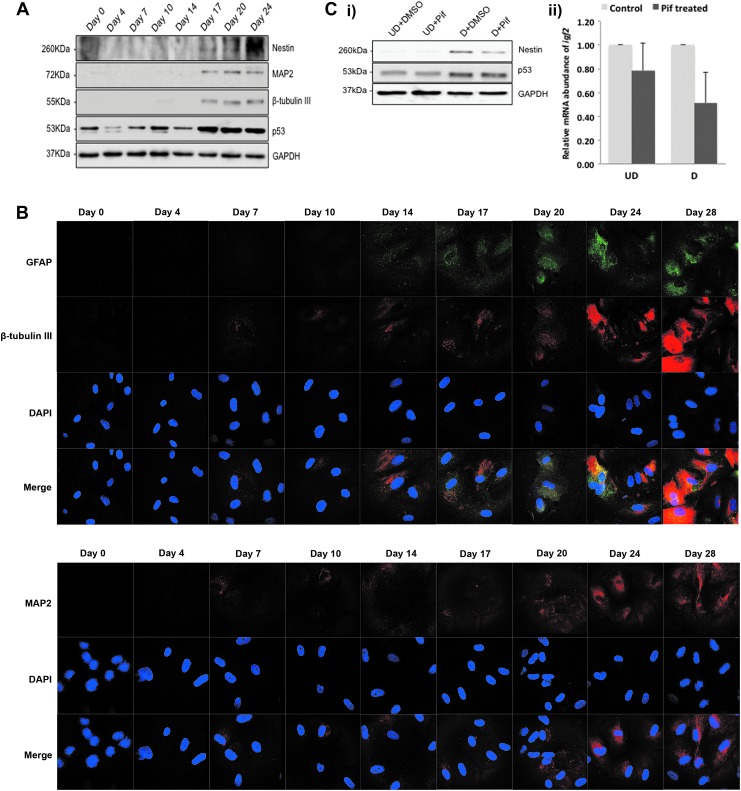
p53 is induced during neural differentiation in hAFS cells. **(A)** hAFS cells were cultured in differentiation medium for 4, 7, 10, 14, 17, 20, and 24 days. Abundance of p53, Nestin, MAP2, and β-tubulin III was monitored by western blotting. GAPDH was used for loading control. **(B)** hAFS cells were cultured on cover slips in differentiation medium for up to 28 days. At the indicated time points, cells were fixed and stained with antibodies for glial fibrillar acidic protein (*green*), β-tubulin III (*red*), and MAP2 (*red*). The cell nucleus was stained with DAPI (*blue*). **(C)** hAFS cells were cultured in duplicates for 9 days in differentiation medium containing retinoic acid (D) or left undifferentiated for control (UD). Where indicated, cells also received pifithrin-α (Pif; 10 μM f.c.) or the vehicle DMSO. One of the duplicates was used to monitor Nestin as a marker for neural differentiation and p53. GAPDH was used for loading control **(i)**. From the second duplicate, RNA was prepared, transcribed into cDNA, and analyzed by qRT-PCR for expression of *igf2*. Abundance of *igf2* mRNA was normalized by determining the abundance of the housekeeping gene β-actin. Relative abundance of *igf2* mRNA in the cells with control siRNA was set to 1. The graph shows mean values and experimental variation of two independent experiments **(ii)**. DMSO, dimethylsulfoxide. Color images available online at www.liebertpub.com/scd

We monitored changes in p53 abundance on days 0, 4, 7, 10, 14, 17, 20, and 24 (Fig. 3A). To follow the process of differentiation of these cells, we also monitored abundance of the neuronal differentiation markers Nestin, MAP2, and β-tubulin III (Fig. 3A). As shown in Fig. 3A, p53 was expressed at much higher levels from day 17 onward. This was also the time when Nestin, MAP2, and β-tubulin III were expressed (Fig. 3A). To confirm the neuronal differentiation seen on western blot, we performed immunostaining against MAP2, β-tubulin III, and GFAP. The expression of these neuronal differentiation markers were seen from day 7 and increased further until day 28 (Fig. 3B).

To determine whether differentiation of hAFS cells depended on p53, we inhibited p53 with pifithrin-α, a molecule that blocks the transcriptional activity of p53. We added 10 μM pifithrin-α to the hAFS cells, a concentration reported earlier to be suitable for ES cells [37], and differentiated the cells with all-*trans* retinoic acid. On the ninth day of differentiation, we harvested the cells and prepared RNA and protein lysates.

As shown in Fig. 3Ci, treatment with pifithrin- α reduced the amount of Nestin in comparison to vehicle-treated cells. This reduction in the expression of Nestin is an indication of reduced differentiation. The signal for Nestin was, however, not completely gone (Fig. 3Ci). We also monitored p53 levels. p53 was induced in the differentiated cells in comparison to the undifferentiated cells (Fig. 3Ci). To determine if pifithrin-α blocked p53's transcriptional activity under our condition, we monitored the mRNA abundance of *igf2*, which we have shown before to be regulated by p53 (Fig. 2A). As shown in Fig. 3Cii, pifithrin-α reduced *igf2* expression by 20% in undifferentiated hAFS cells and by 50% in differentiated hAFS cells. This result shows that pifithrin-α indeed reduced p53's transcriptional activity (Fig. 3Cii).

### p53 is activated in hAFS cells after DNA damage

One crucial function of p53 is to induce cell cycle arrest and apoptosis after DNA damage [30]. In response to DNA-damaging agents, both the levels and activity of p53 increase [38,39]. However, nothing is known about the regulation and function of p53 in hAFS cells. In response to DNA damage, mouse ES cells activate p53 that then binds to the promoters of its canonical target genes, including *bax* and *puma*, resulting in apoptosis [7,40].

**FIG. 4.**
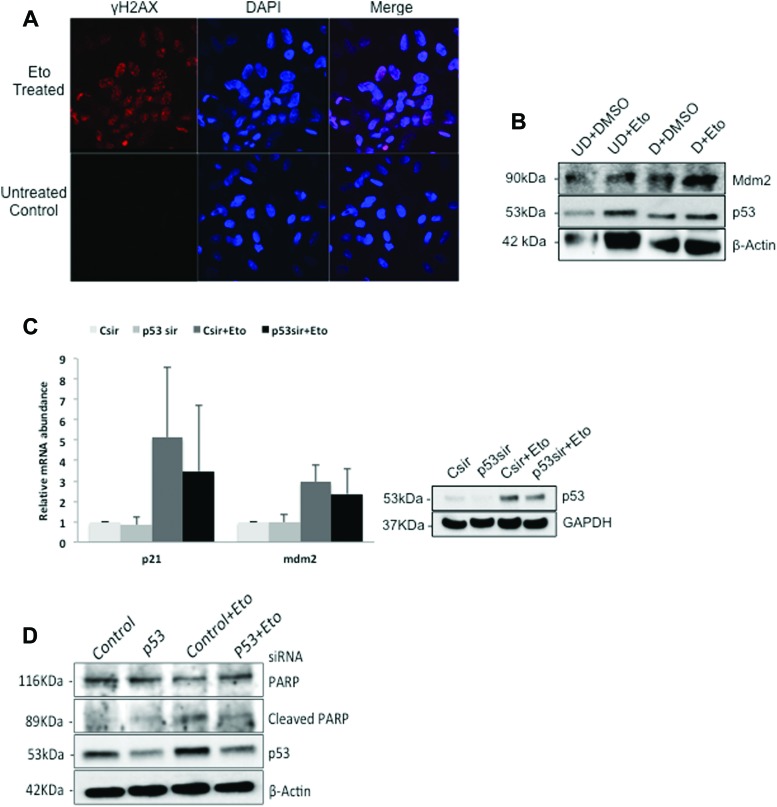
p53 and its target genes are activated in hAFS cells after DNA damage. **(A)** hAFS cells were cultured in chamber slides and treated with 50 μM etoposide for 20 h (Eto treated), or left untreated for control. The cells were fixed and stained with an antibody directed against γ-H2AX (*red*). The cell nucleus was stained with DAPI (*blue*). **(B)** Undifferentiated hAFS cells were treated with 50 μM etoposide for 19 h (UD + Eto), or with DMSO for control (UD + DMSO). Some of the hAFS cells were differentiated for 14 days and then treated with 50 μM etoposide for 20 h (D + Eto), or with DMSO for control (D + DMSO). The cells were harvested and subjected to western blotting to monitor the abundance of p53 and Mdm2. β-actin was used for loading control. **(C)** hAFS cells were transfected in duplicates with a siRNA targeted against p53 (p53sir) or with a control siRNA (Csir). Forty-eight hours after transfection, cells were treated with etoposide (50 μM f.c.) for further 20 h. Cells were harvested and one of the duplicates was used to monitor p53 abundance by western blotting. GAPDH was used for loading control. From the second duplicate, RNA was purified and transcribed into cDNA. The cDNA was analyzed by qRT-PCR for the abundance of p21 and mdm2. Abundance of specific cDNAs was normalized by determining the abundance of the housekeeping gene β-actin. Relative abundance of the specific RNA in the cells with control siRNA was set to one. The graph shows mean values and standard deviations of three independent experiments. **(D)** hAFS cells were transfected with a siRNA targeted against p53 or with a control siRNA for 2 days. Thereafter, some of the cells were treated with etoposide (50 μM f.c.) for 20 h. Cells were lysed and analyzed for PARP, for cleaved PARP, and for p53 abundance. β-actin was used for loading control. PARP, poly ADP-ribose polymerase. Color images available online at www.liebertpub.com/scd

We wondered whether this is also the case for hAFS cells. Therefore, we treated the cells with the DNA-damaging agent etoposide, a topoisomerase inhibitor that induces double-strand breaks [41], and performed immunofluorescence staining to monitor the damage. γ-H2AX, a marker for DNA double-strand breaks, revealed a significant increase in DNA damage in etoposide-treated cells (Fig. 4A). After having confirmed that DNA damage is induced by etoposide in hAFS cells, we performed western blotting to monitor p53 abundance and activity.

To investigate if undifferentiated and differentiated hAFS cells respond differently to DNA damage, we treated both undifferentiated and differentiated hAFS cells with etoposide. As shown in Fig. 4B, treatment of undifferentiated and differentiated hAFS cells with etoposide was accompanied by a strong increase in the amount of the p53 protein. Along with the induction of p53, levels of the p53 target gene Mdm2 were increased too. Overall, we did not find a strong difference in the reaction of differentiated and undifferentiated hAFS cells to etoposide (Fig. 4B).

We next wondered whether the presence of p53 might be required for a normal DNA damage response in hAFS cells. To address this question, we downregulated p53 by employing a siRNA targeted against p53 and treated the cells with etoposide to induce DNA damage. We then investigated the expression of the endogenous p53 target genes *mdm2* and *p21*.

Upon p53 downregulation, p21 mRNA levels were slightly decreased. Treatment with etoposide resulted in about fivefold induction of p21 RNA in cells that had been transfected with a control siRNA. In cells treated with a siRNA that was targeted against p53, this induction was strongly reduced (Fig. 4C). In contrast to *p21*, constitutive expression of *mdm2* was not affected by downregulation of p53. Treatment of hAFS cells with etoposide resulted in a more than threefold increase in mdm2 RNA in cells that had been transfected with a control siRNA. This increase was, however, clearly smaller when p53 was downregulated.

p53 is an important mediator of the DNA damage response and activation of p53 leads to the activation of caspases and induction of apoptosis [42–45]. An earlier report indicated that in undifferentiated ES cells, p53 checkpoint pathways are compromised [19]. We therefore wondered whether p53 is able to control events downstream of DNA damage in hAFS cells. One of the targets of caspase 3 is PARP (Poly ADP-ribose polymerase). Caspase 3 cleaves the 116 kDa full-length PARP protein into an 89 and 24 kDa form [46,47]. We downregulated p53 in hAFS cells, treated the cells with etoposide, and monitored PARP cleavage by western blotting. As shown in Fig. 4D, cleavage of PARP was increased after DNA damage and this increase was lower when p53 was downregulated (Fig. 4D). This result shows that p53 is actively involved in the DNA damage response of undifferentiated hAFS cells.

## Discussion

The demand for establishing stem cell therapy still continues, but with new findings showing that ES cells frequently harbor mosaic alterations and that p53 is frequently mutated in human ES cell lines [48,49], it becomes increasingly challenging to identify a stem cell type that can be used for transplantation into human bodies.

Ideally, the potential candidate should be easy to obtain, have a high proliferation rate in culture, show broad plasticity, and most importantly, be devoid of any ethical issues. hAFS cells possess all these characteristics and fit perfectly as the ideal stem cell type for therapeutic applications. Most importantly, hAFS cells do not form tumors after transplantation into mice and have properties similar to ES cells [13,50]. P53 is an important tumor suppressor protein and therefore it is very important to clarify whether p53 is functional in hAFS cells before they are used in therapy.

We first investigated the localization of p53 in hAFS cells and observed that the majority of the p53 protein is nuclear. This result is consistent with an earlier report on murine ES cells where p53 also has been found in the nucleus of stem cells [14]. We further found that p53 protein levels are relatively stable and do not change with increasing passage numbers. This finding is consistent with previous reports about p53 mRNA levels in these cells [21,23].

p53 is known to be an antiproliferative protein. That is also why p53 mutations are associated with tumor growth in many cancers [51]. We therefore monitored the influence of p53 on the proliferation rate of hAFS cells. Most interestingly, we observed no difference in the proliferation capacity between control cells and cells where p53 was downregulated. This is consistent with an earlier report on murine ES cells where the antiproliferative activity of p53 was also compromised [14]. The suppression of the antiproliferative activity of p53 in hAFS cells prompted us to check for the regulation of some of the noncanonical target genes of p53 that were regulated by p53 in ES cells, including *igf2* and *c-jun* [14].

Although we observed a repression of *c-jun* by p53 in hAFS cells like Yan and co-workers in ES cells, we found that *igf2* mRNA levels were induced by p53 in hAFS cells, while they were repressed by p53 in ES cells [14]. c-Jun, which is encoded by the *c-jun* gene, is a proto-oncogene that achieves its growth-promoting function by heterodimerizing with c-Fos and binding to AP-1 responsive elements in promoters of their target genes, and through repression of the tumor suppressor genes *p53*, *p21*, and *p16* [52]. c-jun has been shown to directly bind and act as an active transcriptional repressor of the p53 promotor [53]. Interestingly, p53 associated with GC-rich regions around the transcriptional start site of *c-jun* in murine ES cells [14], a property that has been described for mutant p53 in differentiated cells [54].

Similar to *c-jun*, *igf2*, which is a maternally imprinted gene that is involved in development, is also a proto-oncogene and a target of p53 that is frequently overexpressed in tumors [55,56]. Surprisingly, although downregulation of p53 strongly induced *igf2* mRNA, we did not see a reduction in *igf2* mRNA after overexpression of p53. The reason for this inconsistency between overexpression and downregulation is unclear. Interestingly, we found expression of *igf2* in cells with a female karyotype, but not in cells with a male karyotype. Eventually, *igf2* expression is not required in male cells. This would be in line with a previous observation where females were strongly dependent on *igf2*, while males were viable also when the *igf2* gene was genetically deleted [34].

We furthermore observed that p53 was strongly induced in hAFS cells as differentiation progressed. Moreover, blocking the transcriptional activity of p53 with pifithrin-α caused a reduction in the amount of Nestin. This result indicates that p53 contributes to the differentiation of hAFS cells. The increase in p53 abundance upon differentiation is in contrast to other reports on ES cells where a significant reduction in the amount of p53 was observed during differentiation [5,15,24,57].

p53 is crucial for the DNA damage response [58]. In our experiments with hAFS cells, p53 also became activated after DNA damage. Its levels were increased and target genes, including *p21* and *mdm2*, were induced. Moreover, we found that cleavage of PARP in response to DNA damage occurred in hAFS cells at least, in part, in a p53-dependent manner. As we did not test other DNA-damaging agents in our study, we cannot comment if hAFS cells respond to all DNA-damaging agents or whether the response is specific to only some adverse agents.

Amniotic fluid contains cells of mixed populations derived from fetus and amnion. Thus a donor-to-donor heterogeneity exists, which might influence not only their proliferation and differentiation capabilities but also their response toward DNA damage. We performed the majority of our experiments with one hAFS cell line. Therefore, similar experiments on more hAFS cell lines that have been established from different donors need to be undertaken to rule out any donor heterogeneity for the regulation of p53's activities.

In conclusion, our studies reveal that p53 is active in hAFS cells. Furthermore, we observed some differences in the activity of p53 in hAFS cells when compared to ES cells. Thus, p53 abundance and activity cannot be generalized in all types of stem cells. This has also been highlighted by the variation of p53 expression in different MSC types [23]. hAFS cells could be potentially useful tools for stem cell therapy, but pertaining to the heterogeneity they possess due to the diversity of the donors, it is very important to investigate the cells in great detail before they enter into therapy.

## Supplementary Material

Supplemental data
