# Supplementary material for: p53 Is Active in Human Amniotic Fluid Stem Cells
Source: Stem Cells Dev. 2018 Oct 25;27(21):1507–17. doi: 10.1089/scd.2017.0254 (PMC6209428; doi:10.1089/scd.2017.0254)
Supplement: Supplemental data [file Supp_Data.pdf]

Supplementary Data

Materials and Methods

Antibodies

Fluorescein isothiocyanate (FITC)-conjugated anti-CD13, anti-CD44, anti-CD45, anti-CD105, and anti-CD166 antibodies and the phycoerythrin (PE)-conjugated anti-CD29 antibody were purchased from Ancell. The FITC-conjugated anti-CD14 and the PE-conjugated CD133 antibodies were obtained from Miltenyi Biotec. The FITC-conjugated antibodies anti-CD90 and anti-SSEA4, the PE-conjugated antibodies anti-HLA-DR, anti-CD73, anti-OCT3/4, and anti-CD146, the Alexa488-conjugated antibody anti-Sox2, anti HLA-ABC, and the allophycocyanin-conjugated antibody anti-CD117 were from Becton Dickinson. The FITC-conjugated antibody anti-CD144 was from Acris Antibodies and the PE-conjugated antibody anti-CD34 was from Beckman Coulter. The anti-hTERT antibody was from Calbiochem and the FITC-conjugated anti rabbit secondary antibody was from Jackson Immuno Research Laboratories.

Immunophenotyping of hAFS cells

Analysis of the expression of cell surface and intracellular protein markers of hAFS cells was performed as previously described [S1,S2]. Briefly, 5×10^5 cells were incubated with 100 μL 20 mM ethylenediaminetetraacetic acid at 37°C for 10 min. Three millimeters washing buffer (0.1% sodium azide, and 0.5% bovine serum albumin in phosphate-buffered saline) was added, the cells were collected by centrifugation at 400*g* for 8 min at 4°C, and the supernatant was discarded.

For staining of the intracellular proteins, the cells were permeabilized by suspending the cell pellet in 1 mL Perm 2 (Becton Dickinson) and incubating them for 10 min at room temperature in the dark. The cells were collected by centrifugation and the supernatant was discarded. The cell pellets were suspended in 100 μL washing buffer containing the recommended amount of surface antibody and incubated for 30 min at 4°C in the dark. The cells were then washed with washing buffer and centrifuged at 400*g* for 8 min at 4°C. The supernatant was discarded and the cell pellet was suspended in 0.5% paraformaldehyde and incubated for 5 min at room temperature. The cells were washed and excess of buffer was discarded, and the cells were kept at 4°C in the dark until they were analyzed using a FACSCanto™ II flow cytometer (BD Biosciences) and the FACDiva v6.1.3 software (BD Biosciences).

Quality control was performed by calibrating the machine regularly with Rainbow Calibration Particles (Becton Dickinson Biosciences). Debris was excluded from the analysis through gating on the morphological parameters on an SSC-A/FSC-A dot-plot. 20,000 events on the morphological gate were recorded for each sample. Nonspecific fluorescence signals were deducted by using isotype control antibodies. All antibodies were titrated under assay conditions. Optimal photomultiplier voltages were established for each channel. The results were analyzed using the FlowJo™ software (Tree Star, Inc.). The mean fluorescence intensity (MFI) ratio was calculated by dividing the MFI of positive events by the MFI of negative events. The cutoff of MFI ratio positivity was <2.0.

Supplementary References

S1. D'Alimonte I, A Lannutti, C Pipino, P Di Tomo, L Pierdomenico, E Cianci, I Antonucci, M Marchisio, M Romano, *et al.* (2013). Wnt signaling behaves as a "Master regulator" in the osteogenic and adipogenic commitment of human amniotic fluid mesenchymal stem cells. Stem Cell Rev 9:642–654.

S2. Pipino C, P Di Tomo, D Mandatori, E Cianci, P Lanuti, MB Cutrona, L Penolazzi, L Pierdomenico, E Lambertini, *et al.* (2014). Calcium sensing receptor activation by calcimimetic r-568 in human amniotic fluid mesenchymal stem cells: correlation with osteogenic differentiation. Stem Cells Dev 23:2959–2971.

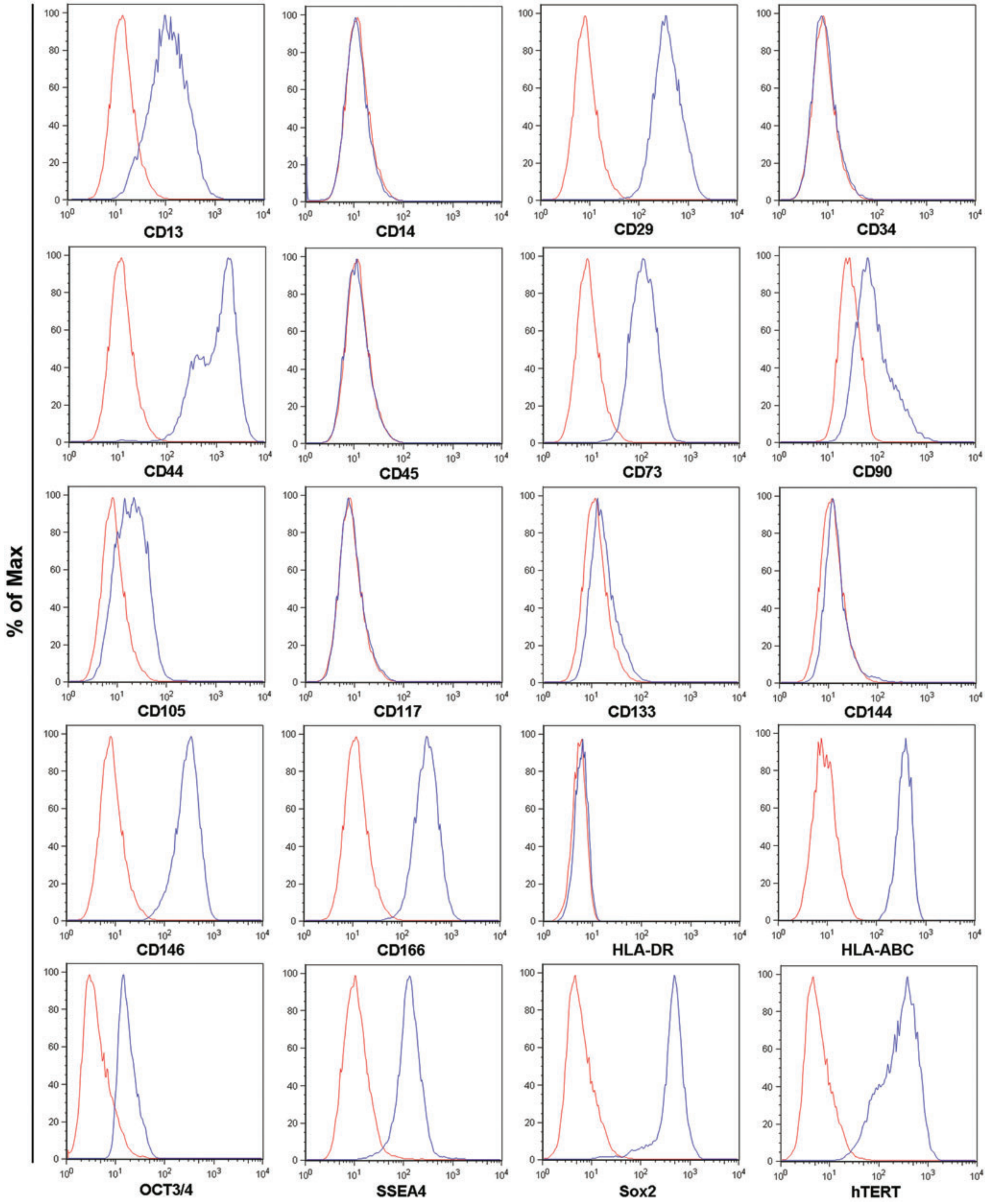

SUPPLEMENTARY FIG. S1. Characterization of hAFS cells. Surface antigen and intracellular marker expression profiles for CD13, CD14, CD29, CD34, CD44, CD45, D73, CD90, CD105, CD117, CD133, CD144, CD146, CD166, HLA-ABC, HLA-DR, OCT3/4, SSEA-4, Sox-2, and h-TERT. The *blue curves* show expression of the individual antigen. The *red curves* represent the signal for the isotype control of the respective antibody. hAFS, human amniotic fluid stem.

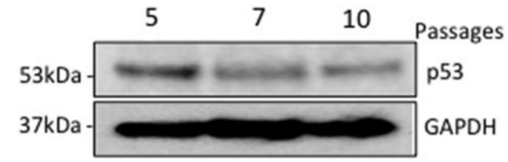

SUPPLEMENTARY FIG. S2. p53 abundance at early and late passages of hAFS cells. hAFS cells were harvested after passage 5, 7, and 10 and analyzed for expression of p53 by western blotting. Detection of GAPDH was used for loading control.

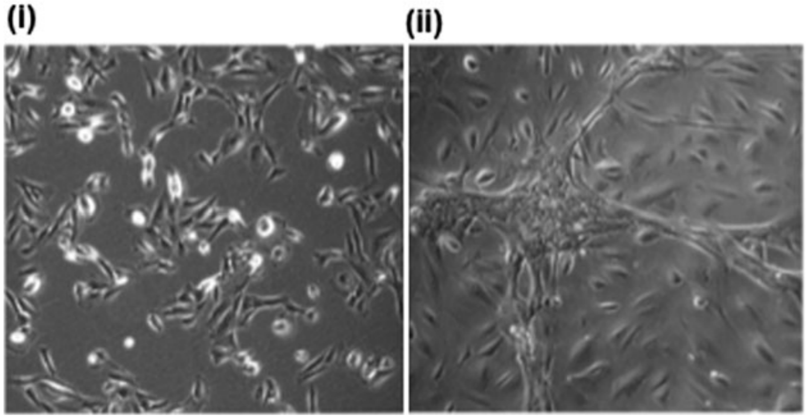

SUPPLEMENTARY FIG. S3. Morphological changes on neural differentiation in hAFS cells. hAFS cells were cultured in neural differentiation medium supplemented with retinoic acid for 14 days and analyzed by microscopy. **(i)** undifferentiated hAFS cells, **(ii)** differentiated hAFS cells.

Supplementary Table S1. Expression of Surface and Intracellular Markers of Human Amniotic Fluid Stem Cells

Antigens	*Phenotype*	*MFI ratio ± SD*
CD13	±	4.1 ± 1.7
CD14	−	1.1 ± 1.0
CD29	+++	65.3 ± 15.3
CD34	−	1.2 ± 0.1
CD44	+++	55.5 ± 13.2
CD45	−	1.3 ± 0.2
CD73	++	18.2 ± 5.4
CD90	+	8.2 ± 2.3
CD105	±	2.9 ± 0.8
CD117	−	1.1 ± 0.3
CD133	−	1.2 ± 0.2
CD144	−	1.3 ± 0.1
CD146	++	31.2 ± 11.4
CD166	+	16.7 ± 4.3
HLA-ABC	+	30.9 ± 9.8
HLA-DR	−	1.1 ± 0.1
OCT3/4	+	3.9 ± 0.9
SSEA-4	++	45.6 ± 12.3
Sox-2	+++	78.4 ± 19.3
h-TERT	+++	71.5 ± 16.3

−, negative expression; ±, low expression; +, moderate expression; ++, positive; +++, high expression; MFI, mean fluorescence intensity.
